# Evolutionary pathways for deep-sea adaptation in marine planktonic *Actinobacteriota*

**DOI:** 10.3389/fmicb.2023.1159270

**Published:** 2023-05-10

**Authors:** Juan J. Roda-Garcia, Jose M. Haro-Moreno, Mario López-Pérez

**Affiliations:** Evolutionary Genomics Group, División de Microbiología, Universidad Miguel Hernández, Alicante, Spain

**Keywords:** *Acidimicrobiales*, *Actinobacteriota*, oxygen minimum zone, deep ocean, WhiB, nitrate reductase, dissolved organic matter, cytochrome P450 monooxygenases

## Abstract

The deep ocean, one of the largest ecosystems on earth, is dominated by microorganisms that are keystones in the regulation of biogeochemical cycles. However, the evolutionary pathways underlying the specific adaptations required (e.g., high pressure and low temperature) by this unique niche remain understudied. Here, we analyzed the first representatives belonging to the order *Acidimicrobiales*, a group of marine planktonic *Actinobacteriota*, that specifically inhabits the aphotic zone of the oceanic water column (>200 m). Compared with their epipelagic counterparts, deep-sea representatives showed the same evolution in genome architecture with higher GC content, longer intergenic spaces as well as higher nitrogen (N-ARSC) and lower carbon (C-ARSC) content in encoded amino acid residue side chains consistent with the higher nitrogen concentration and lower carbon concentration in deep waters compared to the photic zone. Metagenomic recruitment showed distribution patterns that allowed the description of different ecogenomic units within the three deep water-associated genera defined by our phylogenomic analyses (UBA3125, S20-B6 and UBA9410). The entire genus UBA3125 was found exclusively associated with oxygen minimum zones linked to the acquisition of genes involved in denitrification. Genomospecies of genus S20-B6 recruited in samples from both mesopelagic (200–1,000 m) and bathypelagic (1000–4,000 m) zones, including polar regions. Diversity in the genus UBA9410 was higher, with genomospecies widely distributed in temperate zones, others in polar regions, and the only genomospecies associated with abyssal zones (>4,000 m). At the functional level, groups beyond the epipelagic zone have a more complex transcriptional regulation including in their genomes a unique WhiB paralog. In addition, they showed higher metabolic potential for organic carbon and carbohydrate degradation as well as the ability to accumulate glycogen as a source of carbon and energy. This could compensate for energy metabolism in the absence of rhodopsins, which is only present in genomes associated with the photic zone. The abundance in deep samples of cytochrome P450 monooxygenases associated with the genomes of this order suggests an important role in remineralization of recalcitrant compounds throughout the water column.

## Introduction

The phylum *Actinobacteriota*, according to the recent Genome Taxonomy Database (GTDB) classification (Parks et al., 2018), (or *Actinobacteria*), comprises a group of microorganisms widely studied because they are producers of several bioactive substances with biotechnological potential. Although originally defined as a group of typically soil bacteria, with a high GC and a diverse morphology, this group of microbes has also been found in aquatic environments. For instance, the actinomycete genus *Salinispora*, which inhabits marine sediments, has been extensively studied since it is a rich source of secondary metabolites (Fenical and Jensen, 2006). The study of genomic plasticity of this type of natural product biosynthetic gene clusters suggests that it is an important driver of bacterial diversification and may contribute to the cosmopolitan distribution of this genus (Penn et al., 2009; Ziemert et al., 2014; Letzel et al., 2017). Culture-independent methods have revealed that members of the phylum *Actinobacteriota* are among the most abundant and widespread groups in freshwater habitats (Ghai et al., 2014). At the same time, they are also a widespread pelagic phylum in the oceans, representing up to 5% of the total bacterial population in oligotrophic waters (Morris et al., 2005, 2012; Treusch et al., 2009).

Described for the first time by PCR analysis of 16S rRNA gene sequences from seawater (Fuhrman et al., 1995), the marine pelagic *Actinobacteriota* was later divided into two groups (i) “*Candidatus*” Actinomarinales (Ghai et al., 2013; López-Pérez et al., 2020) and (ii) the *Acidimicrobiales* (Mizuno et al., 2015). Members of the “*Ca.* Actinomarinales” are examples of streamlined bacteria, similar to the alphaproteobacterial SAR11 clade, with a low GC content (33%), small genome size, estimated to be close to 1 Mb, and small intergenic spacers (2 bp) (López-Pérez et al., 2020). Remarkably, their volume size of 0.013 μm^3^, the lowest described so far, makes the members of this group the smallest free-living marine microbes (Mizuno et al., 2015). Conversely, members of the order *Acidimicrobiales* exhibit larger genomes (1.7–2.3 Mb) and have a moderate to high GC content, between 40 to 50%.

Despite the notable differences in genome size and GC content, both groups share similar metabolic traits. Both are photo-chemoorganotrophs, containing transporters for sugars and amino acids, enzymes involved in the glycolysis, pentose phosphate and TCA pathways, and the light-harvesting rhodopsin (Ghai et al., 2013; Mizuno et al., 2015). However, their rhodopsins are phylogenetically distant. While the rhodopsins encoded within the marine *Acidimicrobiales* cluster together with the freshwater counterparts (Mizuno et al., 2015), the rhodopsins within the “*Ca.* Actinomarinales” form a new group named Marine Actinobacterial Clade Rhodopsins (MAC-rhodopsins; Ghai et al., 2013). The larger genome size of the *Acidimicrobiales* allows them to use several other nutrients (DMSP, C2 compounds or CO, among others) as sources of carbon and energy (Mizuno et al., 2015). These two groups co-occur in temperate and tropical waters, although only *Acidimicrobiales* have been detected from polar samples (Alonso-Saez et al., 2012). In temperate waters, such as those of the Mediterranean Sea, these groups can be found throughout the photic zone of the water column, both in the stratification period (summer) and in winter, when the water column is mixed (Haro-Moreno et al., 2018). However, while “*Ca.* Actinomarinales” showed a predilection for the upper layers of the epipelagic zone, members of *Acidimicrobiales* were found more prevalent in the deep photic zone (around the deep chlorophyll maximum; Mizuno et al., 2015; López-Pérez et al., 2020).

Recently, we analyzed variations in microbial communities along a depth profile from the epipelagic to the mesopelagic zone in the Mediterranean Sea (Haro-Moreno et al., 2018). From a sample obtained at 1,000 m, we were able to recover several metagenome-assembled genomes (MAGs). Taxonomic classification based on the GTDB revealed that three of these new MAGs were affiliated with three different genera within the same family, family MedAcidi-G1 (order *Acidimicrobiales*). This family also includes two of the first representative MAGs of this order (MedAcidi-G1 and MedAcidi-G2B) previously described in Mizuno et al. (2015) which are also different genera. However, while these new MAGs were reconstructed from a mesopelagic sample, the others have only been found in epipelagic zones. The presence of two different lifestyles within the same family provides the ideal framework for studying in detail how evolutionary pressure may drive different adaptations to the environment in closely related *Actinobacteriota* groups. Therefore, in this study, we have collected all the genomic diversity of this family available in public databases (available up to July 2021) and analyzed the phylogeny, biogeography, and metabolism by comparative genomics.

## Materials and methods

### Phylogenomic and genomic characteristics

To obtain the whole genomic diversity of MedAcidi-G1 family, all available genomes were downloaded from the National Center for Biotechnology Information (NCBI; Agarwala et al., 2016) and the Integrated Microbial Genomes system (IMG; Markowitz and Kyrpides, 2007; available up to July 2021). The degree of completeness and contamination of the genomes were estimated using CheckM v1.1.2 (Parks et al., 2015), and only those with completeness >50% and contamination <5% were kept. ANI between pairs of genomes was calculated with the PYANI software (Pritchard et al., 2016) and we dereplicated all these genomes to an identity of ANI >99% using dRep software (Olm et al., 2017). The final dataset of 136 genomes (Supplementary Table S1) was used to establish the phylogenomic classification using Phylophlan 3.0 (Segata et al., 2013) with the genomes of “*Ca.* Actinomarinales” (López-Pérez et al., 2020) as an outgroup. For each genome, the GC content and the size of the intergenic space were calculated by using Gecee program from the EMBOSS package (Rice et al., 2000) and an in-house Perl script, respectively. N-ARSC and C-ARSC values were estimated using the script described in Mende et al. (2017).

### Metagenomic recruitment

To determine the ecological distribution of the different MedAcidi-G1 genomes in the global ocean, we performed metagenomic recruitment using samples from *Tara* Oceans expedition (Sunagawa et al., 2015), *Tara* Oceans Polar Circle (Tara Oceans Consortium, C., and Tara Oceans Expedition, P, 2017), Geotraces (Biller et al., 2018), Polar biomes (Zhang et al., 2020), Tasman sea (PRJNA385736), HOT ALOHA metagenomic time and depth series (Mende et al., 2017), Malaspina expedition (Duarte, 2015), Mediterranean Sea (Haro-Moreno et al., 2017, 2018; López-Pérez et al., 2017), Red Sea time and depth series (Haroon et al., 2016) and western subarctic Pacific (PRJNA398459). Metagenomic reads were trimmed using Trimmomatic v0.36 (Bolger et al., 2014) and only those readings with a Phred score of ≥30, that were ≥ 50 bp long, and without ambiguity were used for recruitment analysis. To determine the presence of a genome in a sample we required that >70% of the genome was covered with a read identity cutoff of ≥98% (length ≥ 50 bp) and that the value of reads per kilobase of genome and metagenome gigabase (RPKG) was at least five.

### NarG sequence retrieval and phylogenetic analysis

All sequences annotated as respiratory nitrate reductase alpha subunit (NarG) were downloaded from NCBI (available up to December 2022) and manually curated before aligning using MUSCLE (Edgar, 2004). Hidden Markov models (HMMs) of these sequences were built using hmmbuild (Finn et al., 2011). Putative NarG sequences from *Tara* Oceans metagenomes (TARA_100 and TARA_137) were detected via HMMER hmmscan (Finn et al., 2011). A maximum-likelihood phylogenetic tree was built using IQ-TREE2 (Nguyen et al., 2015) with 1,000 ultrafast bootstraps and the option -m MFP to assess the best-fitted model automatically.

### Functional classification

Coding DNA sequences for each genome were predicted using Prodigal (Hyatt et al., 2010). To perform a functional comparison, we clustered all the sequences belonging to each genus using CD-HIT v4.8.1 (Huang et al., 2010) with a minimum percentage of identity of 70% and a coverage of at least 50%. The resulting clusters were annotated against the SEED subsystem database (Overbeek et al., 2005) using DIAMOND v0.9.34 (Buchfink et al., 2015; blastp option, top hit, ≥ 50% identity, ≥ 50% alignment length, *E*-value <10^−5^) and KEGG (Kyoto Encyclopedia of Genes and Genomes) using the BlastKOALA V.2.2 tool (Kanehisa et al., 2016). In addition, an in-depth functional classification of the cytochrome P450 monooxygenases coded proteins was performed using the Cytochrome P450 Engineering Database v6.0 (CYPED; Sirim et al., 2009). To sum up, for each family described and available in the CYPED database (available up to December 2022) a HMM database was constructed using the hmmbuild program of the HMMER package (Finn et al., 2011). Before that, protein sequences were aligned to the database using muscle (Edgar, 2004). Next, the set of clustered proteins from each of the five genera was compared with the CYPED database using hmmscan, and only the resulting hits with an e-value <1e-5 and a bitscore >75 were kept. A similar search was performed against the retrieved proteins from the assembled contigs of two marine metagenomic studies: a depth profile (spanning the depths 15, 30, 45, 60, 75, 90, and 1,000 m) from the Mediterranean Sea (Haro-Moreno et al., 2018) and the Malaspina Expedition, which collected samples from the bathypelagic realm (Duarte, 2015).

## Results

### Phylogenomics

**Figure 1:**
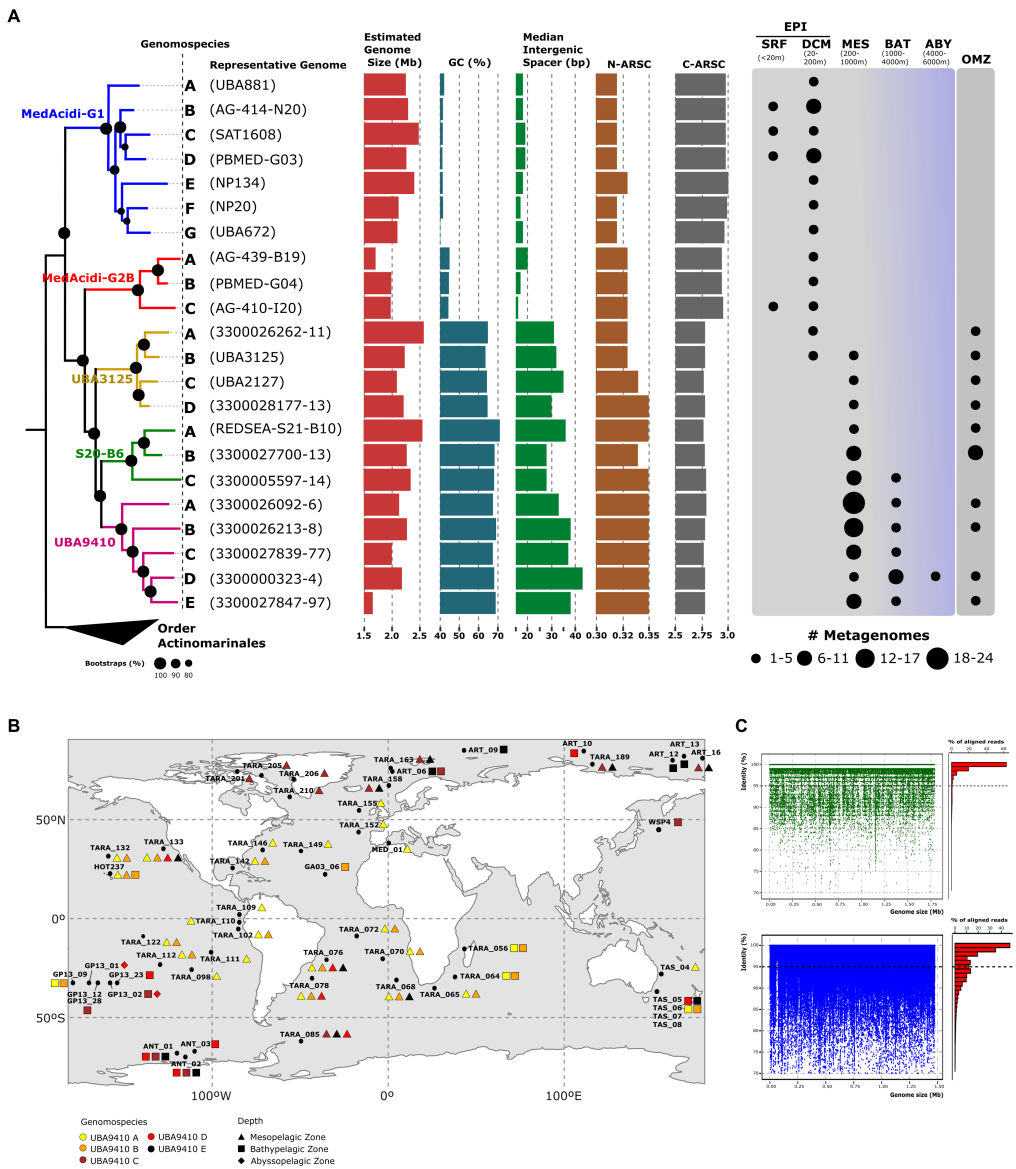
**(A)**: maximum likelihood phylogenomic tree of the MedAcidi-G1 genomospecies. The letters refer to genomospecies and between brackets, we find the most complete genome used as a representative. The branches have been colored according to the genus to which they belong. Genomes of the other order of marine pelagic *Actinobacteriota* “*Ca.* Actinomarinales” were used as outgroups. Bootstrap values are indicated as black circles on the nodes. Genomospecies median for the GC content, estimated genome size, and intergenic spacer together with N-ARSC and C-ARSC, is plotted next to the tree. On the right side, we find the presence of the reference genomes in different metagenomic data sets grouped by depth. The size of the circles represents the number of metagenomes in which the genome was found. SRF (surface); DCM (deep chlorophyll maximum); MES (mesopelagic); BAT (bathypelagic), ABY (abyssopelagic), OMZ (oxygen minimum zone). **(B)**: geographical distribution of the five genomospecies within the genera UBA9410 in different mesopelagic metagenomic datasets described in Supplementary Table S2. The colors refer to the different genomospecies and the geometric shapes to the depth where they recruited (triangle, mesopelagic zone; square, bathypelagic zone; rhombus, abyssopelagic zone). **(C)**: recruitment plots of one representative genome of UBA9410-D genomospecies with different behavior. “Polyclonal” in green and “multispecies” in blue.

First, we collected all the genomic diversity available in the databases corresponding to the MedAcidi-G1 family within the order *Acidimicrobiales* (see Methods). To perform our phylogenomic analysis, we removed all genomes that did not meet the quality criteria to be considered medium-to high-quality draft genomes (>50% completeness and < 5% contamination; Bowers et al., 2017) and then dereplicated all genomes with ANI >99% identity to avoid clonality. Detailed genomic features and metadata of the final dataset of genomes are shown in Supplementary Table S1. In the end, we recovered 136 genomes [130 MAGs and 6 Single-Amplified Genomes; (SAGs)] that were used to perform the phylogenomic tree using the other order of marine pelagic *Actinobacteriota* [“*Ca.* Actinomarinales” (López-Pérez et al., 2020)] as an outgroup. The resulting tree topology revealed five well-defined branches corresponding to five genera according to the divergence obtained in the ANI comparison (>70%; Figure 1A; Supplementary Table S1). Each of the genera has been named according to the classification assigned by the GTDB (S20-B6, UBA9410, UBA3125, MedAcidi-G1, and MedAcidi-G2B).

### Ecological distribution and lifestyle

Next, we sought to delve into the global distribution and vertical distribution patterns of each genera using a global metagenomic dataset (see Methods). The results showed a vertical stratification of the family consistent with the phylogenomic classification. The genera MedAcidi-G1 and MedAcidi-G2B were present in the lower layers of the epipelagic zone (around the deep chlorophyll maximum) but never beyond 150 m. In contrast, the other three genera were mostly found in deep aphotic datasets, but with different recruitment patterns. While the distribution of genus UBA3125 was found to be exclusively associated with oxygen minimum zones (OMZ, oxygen concentration below 5 μmol/l (Kalvelage et al., 2011)) of mesopelagic regions, the genomes of genus S20-B6 also recruited in bathypelagic samples (1000–4,000 m; Figure 1A). The genus UBA9410 showed cosmopolitan distribution and could be detected from mesopelagic to abyssal zones (>4,000 m; Figure 1A).

However, the distribution was not homogeneous for all genomes within each genus. The analysis of the recruitment patterns allowed the differentiation of phylogenetically related ecogenomic units or genomospecies within each genus (Haro-Moreno et al., 2020; López-Pérez et al., 2020; Roda-Garcia et al., 2022). For instance, within the genus S20-B6, we found three genomospecies, referred to as A, B and C. Genomespecies A, made up of MAG reconstructed from the Red Sea that show high recruitment only at the site where the sequences were obtained, suggesting specific environmental selection (Supplementary Table S2). The four samples from the Red Sea where these genomes recruited were collected at 500 m and showed atypical temperature and salinity conditions (21.5°C and 40.5 PSU salinity) as well as low oxygen concentrations (*ca.* 1 μmol/L) compared to the global deep ocean (Haroon et al., 2016). Genomospecies B was detected only in mesopelagic zones belonging to OMZ (Southeast Pacific Ocean and North Indian Ocean) and genomes from genomospecies C recruited in both mesopelagic and bathypelagic zones including polar regions (Figure 1A; Supplementary Table S2). Although all genomes of the genus UBA3125 were restricted to the OMZ, we were able to differentiate four genomospecies. While genomospecies B were detected in northern (TARA_137) and southeastern (TARA_100, TARA_102, and TARA_110) mesopelagic regions of the Pacific Ocean, genomospecies C and D were restricted to the northern Indian (TARA_37, TARA_38, and TARA_39) and Pacific Ocean (TARA_137 and TARA_138), respectively (Table S2). Genomospecies A was only present at station TARA_100 (southeast Pacific Ocean). Among the five genomospecies of the genus UBA9410, A and B showed a more widespread distribution than the other genomospecies in tropical and subtropical waters, being present in the mesopelagic metagenomes of *Tara* expedition from the Atlantic, Pacific and Indian Oceans (Figure 1B; Supplementary Table S2). The distribution of genomospecies C, D and E were preferentially found in higher latitudes, to cold waters above 50°N and 50°S. Only members of the genomospecies D recruited in abyssopelagic samples obtained in the Pacific Ocean during the GEOTRACES GA13 expedition (Figure 1B; Supplementary Table S2).

### Genomic diversity in the genus UBA9410

The wide spatial distribution range as well as throughout the entire water column of the deep aphotic zone of the genomospecies UBA9410-D allowed us to analyze genome-wide diversity patterns using MAG 3300000323–4, reconstructed from a sample at 2,000 m from the North Pacific Ocean, as a reference. To avoid possible bias in recruitment, linear recruitment plots were made in those samples where the genome recruited at least ten reads per kilobase of genome per gigabase (RPKGs) of metagenome and a coverage of >90%. We found the same pattern of diversity in all the samples at the genome level, regardless of latitude or depth. We observed a sequence-discrete population where the reads recruited with a similarity of up to 98% nucleotide identity to the reference genome indicating a polyclonal behavior of the population (Figure 1C). Using those metagenomes, we calculated the average nucleotide identity based on reads (ANIr). The median ANIr value for the UBA9410-D representative was 98.4%, suggesting a low intrapopulation sequence diversity. Because of their abundance in mesopelagic samples from temperate (UBA9410-A) and polar (UBA9410-C) regions, we also examined population-level heterogeneity within these two genomospecies. Using the most complete genome of each genomospecies (based on CheckM (Parks et al., 2015)), the median ANIr was calculated in five different metagenomes where they recruited the most. While genomospecies UBA9410-C showed a value of 98.3%, genomospecies A had a slightly lower median, 97.2%. Therefore, the three genomospecies showed the same intrapopulation sequence diversity. These ANIr values above 97% as well as the linear recruitments (Figure 1C) suggest that in nature several clones of the same genomospecies coexist in the same sample simultaneously (Haro-Moreno et al., 2019). Although this is the most widespread pattern for UBA9410 genomospecies, in a few stations we found another pattern of recruitment where apart from the reference genome, several species (ANI below 95%) grew simultaneously in the same sample (Figure 1C) defined as a “multispecies” behavior (Haro-Moreno et al., 2019).

### *Acidimicrobiales* in oxygen minimum zones

The fact that all the genomospecies from genus UBA3125 recruited in oxygen-deprived regions of the ocean led us to investigate possible metabolic adaptations to low oxygen in these genomes. Metabolic reconstruction was carried out using the KEGG database by clustering all the genes of the genomes belonging to the genus. Interestingly, we detected genes involved in denitrification. In zones of oxygen depletion conditions such as the OMZ, this respiratory pathway allows microorganisms with a microaerophilic or facultative anaerobic metabolism to use nitrate as an electron acceptor instead of oxygen (Pajares and Ramos, 2019). Nitrate is introduced into the cell via the nitrate/nitrite transporter (*nark*) for subsequent reduction to dinitrogen by progressive reductions catalyzed by a nitrate reductase (*narGHJI*), a nitrite reductase (*nirK*), a nitric oxide reductase (*norBC*) and a nitrous-oxide reductase (*norZ*). Except for the nitric oxide reductase all genes were found in UBA3125, as well as the nitrate/nitrite response regulator (*narL*). NarL is a transcriptional regulator involved in changes from aerobic to anaerobic respiration by activating the expression of nitrate reductase genes and repression of other nitrate-regulated respiration genes such as fumarate reductase (Katsir et al., 2015; Yoshida et al., 2015). It has been previously suggested that many microorganisms encode only part of the denitrification pathway (Graf et al., 2014), therefore the results seem to indicate that, in accordance with their distribution in the OMZ, these microbes are able to respire nitrate under low oxygen conditions. Some genomospecies of the other deep-sea genera also recruit in areas of the OMZ, however, the same analysis done for genus UBA3125 revealed that genus S20-B6 only possesses the nitrate/nitrite transporter and the nitrate reductase. In genus UBA9410, only nitrite reductase was found. The lack of one or more genes of the denitrification pathway known as truncated denitrification pathway has been explained by the synergistic action between different taxa coexisting in the same environment (Zumft, 1997; Wallenstein et al., 2006).

**Figure 2:**
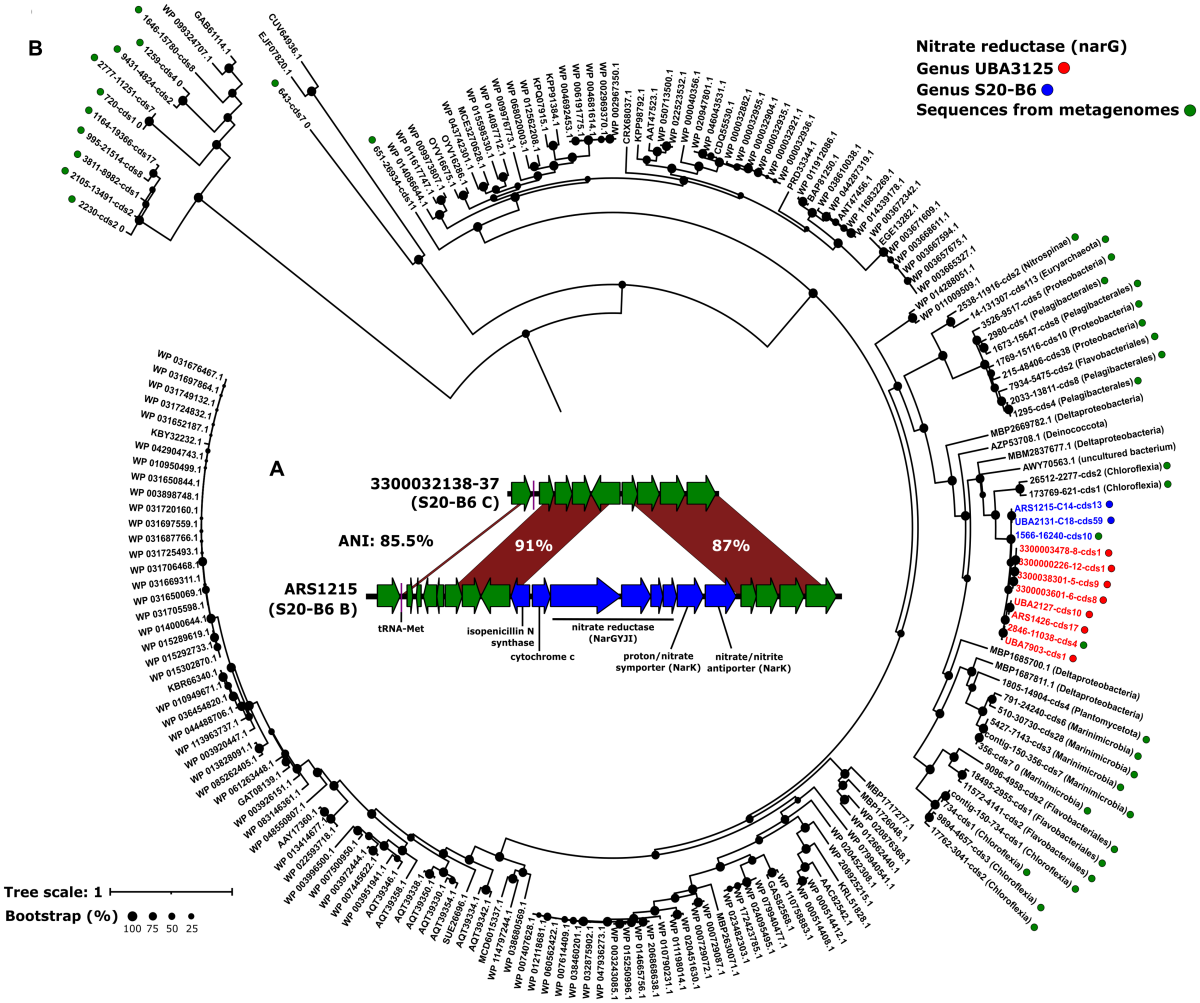
**(A)**: maximum likelihood phylogenetic tree of NarG sequences detected in metagenomes from Eastern Tropical North and South Pacific Oxygen Minimum Zone (TARA_100 and TARA_137). Green dots indicate sequences obtained in the metagenomes. The sequences obtained from the genomes and clustered at 99% identity are shown as blue and red circles for the S20-B6 and UBA3125 genera, respectively. The rest are NCBI reference sequences. Bootstrap values are indicated as black circles on the nodes. **(B)**: schematic representation comparing the genomic island of the gene cluster involved in denitrification.

Genomic comparison between genomes of the genomospecies present (B) and absent (C) in the OMZ within the genus S20-B6 showed that the nitrate reductase was located in a genomic island of 11Kb (Figure 2A). This region contains genes coding not only for nitrate reductase (NarGYJI) but also for two different NarK-like transporters, a proton/nitrate symporter, and a nitrate/nitrite antiporter (Figure 2A). On the island, we also found a c-type cytochrome that could be involved in the respiratory electron transport chain. Then, we analyzed the divergence of the *narG* gene, encoding the alpha subunit of respiratory nitrate reductase, in two OMZ metagenomic samples (Eastern Tropical North and South Pacific; TARA_100 and TARA_137) in which several genomospecies of the genera UBA3125 and S20-B6 recruited. We first obtained all the genetic diversity of this gene in the metagenomes (see Methods) and made a phylogenetic tree (Figure 2B). The resulting tree showed that all MedAcidi-G1 family sequences clustered together (genus UBA3125 and S20-B6) in the same branch. However, we found other nearby sequences that clustered with these sequences. The small size of the recovered contigs made it difficult to compare them, although several of them could be taxonomically assigned as sequences affiliated to the phylum *Chloroflexota*, one of the most abundant groups in the global deep ocean (Morris et al., 2004; Mehrshad et al., 2018).

### Variation in genomic features within family MedAcidi-G1

The differential ecological distribution of the groups within the family could also translate into changes in the genomic architecture of these microbes. Therefore, we compared some general genomic features using as a reference dereplicated genomes. Despite being closely related at the phylogenetic level, genomes associated with deep waters showed a much higher GC content (ranging from 57 to 72%) than the photic zone genera (mean 42.41% [SD, ±1.49]) (Figure 1A; Supplementary Table S1). The wide range of values within the deep-associated genera is due to the fact that the genomes of the OMZ genus (UBA3125) showed values (mean 63.48% [SD, ±2.68]) slightly lower than those of UBA9410 and S20-B6 (mean 66.84% [SD, ±2.55] and mean 69.02% [SD, ±1.62], respectively) (Figure 1A; Supplementary Table S1).

The analysis of the average number of nitrogen (N-ARSC) or carbon (C-ARSC) atoms per side chain of amino acid residues showed significant differences between the two groups. The genomes of microbes from areas beyond the photic zone had a higher N-ARSC (0.344 ± 0.007 versus 0.323 ± 0.003; *p* value <0.01) and a lower C-ARCS (2.73 ± 0.02 versus 2.88 ± 0.02; *p* value <0.01). We also observed a reduction in intergenic spacer size in epipelagic genomes compared to their depth counterparts. The genomes of the genera in the aphotic zone showed values between 24 and 46 bp (mean deep clade 33.54 bp [SD, ±5.15]), while the mean of the genomes in the photic zone was 18.58 bp [SD, ±3.09]). Unlike the other parameters, the difference in the estimated genome size between the depth and surface groups was not statistically significant (*p* value >0.01). The genomes of the genus MedAcidi-G2B had the smallest values with a mean size of 1.95 Mb (SD, ±0.14) and the deep water-associated genus S20-B6 had the largest size with a mean value of 2.38 Mb (SD, ±0.25).

### Metabolic adaptations to the deep ocean

**Figure 3:**
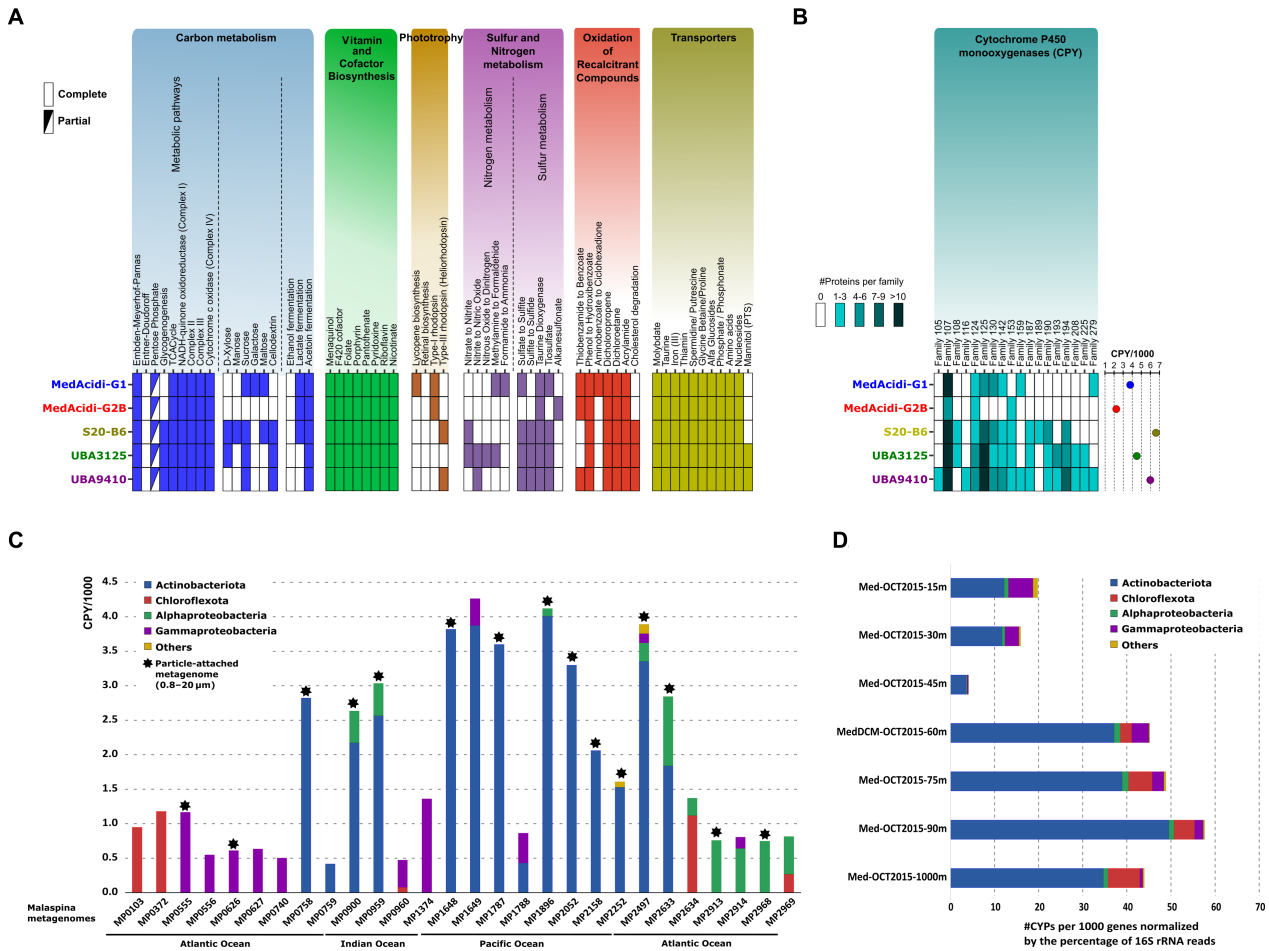
**(A)**: inferred metabolism of the five genera within the MedAcidi-G1 family (order *Acidimicrobiales*) based on the KEGG database. **(B)**: number of cytochrome P450 monooxygenases (CYPs) detected. The circles on the right show the normalized abundance per 1,000 genes. **(C)**: abundance of the CYPs (normalized per 1,000 genes) by phylum in samples of the Malaspina expedition (Acinas et al., 2021). Asterisks indicate samples belonging to the particulate fraction (0.8–20 μm). **(D)**: number of CYPs per 1,000 genes normalized by the percentage of 16S rRNA reads by phylum and depth of the water column at a single site from the western Mediterranean (Haro-Moreno et al., 2018).

*General metabolism of the MedAcidi-G1 family.* Once the variations in genome architecture were analyzed, we wanted to evaluate which metabolic adaptations have allowed adaptation to environments beyond the epipelagic zone in the water column. Despite the different niches in which they are located, we found certain common characteristics in all genera of the family. As already described by Mizuno et al. (2015), the five genera encoded an aerobic heterotrophic metabolism. They encode for the Embden–Meyerhof–Parnas (glycolysis) pathway, the tricarboxylic acid cycle and the electron-transport chain (complexes I to IV; Figure 3A). The number and type of transporters was also similar. All genera encoded the subunits for the acquisition of molybdate, taurine, ferric iron, the vitamin thiamine (B1), general amino acids, spermidine/putrescine, and nucleosides (Figure 3A). In the same way as their counterparts in the epipelagic zone, none of the genera associated with deep waters had genes related to flagellum synthesis and, therefore, they are not motile. In almost all genera, we found enzymes involved in acetoin fermentation and potentially in lactate fermentation due to the presence of lactate dehydrogenase. In addition to the presence of these genes for fermentation, in the deep-associated genera (S20-B6, UBA3125 and UBA9410) we also found others involved in the utilization of nitrate as an electron acceptor which could indicate that they can also grow in environments with low oxygen concentrations, such as the OMZ (see above) or within particles. Besides, the five genera are capable of synthesizing the 20 essential amino acids, as well as the vitamins riboflavin (B2), pyridoxal (B6), nicotinate (B3), pantothenate (B5), folate (B9) (Figure 3A). We also detected the biosynthesis of cofactors menaquinol and 8-hydroxy-5-deazaflavin (also named F_420_), both involved in redox reactions, and the biosynthesis of the heme ring, important for the function of cytochromes (see below).

Genes involved in the sulphur cycle were also present in the genomes of the MedAcidi-G1 family. For instance, all genera encoded for genes responsible for the degradation of taurine to sulfite (taurine dioxygenase), the oxidation of thiosulfate to sulfite (thiosulfate sulfurtransferase), and the reduction of sulphate to sulfite due to the presence of the enzymes 3′-phosphoadenosine 5′-phosphosulphate synthase (PAPSS) and phosphoadenosine phosphosulfate reductase (CysH). However, only the genera of the mesopelagic zone encoded for the sulfite reductase CysJ protein, to assimilate the reduced sulfur into biomass (Figure 3A). Lastly, we only detected the presence of proton pump rhodopsins (Mizuno et al., 2015) in the MedAcidi-G1 and MedAcidi-G2B genera. We also found in S20-B6 and UBA9410 proteins classified as heliorhodopsin. However, a closer inspection of the individual genomes indicated that this protein is not conserved, due to it appeared only in 4% of the genomes affiliated with these two genera. This result contrasts with what has been found in the other marine Actinobacteriota group, *“Ca.* Actinomarinales*,”* on which all genera described encoded a proton-pump rhodopsin and a heliorhodopsin in their genomes (López-Pérez et al., 2020).

*Differences in sugar metabolism.* The comparison of the genetic repertoire of the different groups showed clear distinctions in their nutrient preferences. Regarding the carbohydrate metabolism, the deep-associated genera encoded exclusively genes involved in the glycogen biosynthesis pathway (*glg*ABC), as well as the glycogen phosphorylase *glg*P, thus indicating the accumulation of glycogen inside the cells within the deep *Acidimicrobiales* (Figure 3A). Accumulated glycogen is a source of carbon and energy when glucose is unavailable in the environment. There were also differences in the sugar uptake and degradation. Genera S20-B6 and UBA3125 seemed capable of degrading the monosaccharide xylose, while all three deep water-associated genera were able to degrade the cellulose derivative cellodextrin and sucrose (Figure 3A). The genus S20-B6 seems to be the most versatile of all in the utilization of sugars since it is the only one that also has the genes to potentially degrade mannose and maltose. Conversely, only the genus MedAcidi-G1 encoded the enzymes responsible for the galactose transformation toward glucose.

*Role in the (recalcitrant) organic carbon remineralization.* The inspection of the annotated proteins resulted in the identification of enzymes involved in the degradation of halogenated carbon compounds, such as dichloropropene and dichloroethane, conserved within the five genera. In addition, enzymes for the degradation of aromatic compounds were also detected. Genes for the degradation of thiobenzamide to benzoate were detected within MedAcidi-G1, MedAcidi-G2B and UBA9410, while the degradation of aminobenzoate was only detected within the genomes of MedAcidi-G1. Genes for the degradation of phenol were found within all genera. However, cholesterol degradation was only present in the genera of the aphotic zone.

Interestingly, we detected a significant number of cytochrome P450 monooxygenases (CYPs) within the order *Acidimicrobiales*. This group of enzymes has been found in organisms from all kingdoms of life (Nelson, 2018; Lamb et al., 2019) and plays a role in catalyzing many types of reactions, such as hydroxylation, epoxidation, oxidation, dehalogenation, and dehydrogenation (Urlacher and Schmid, 2002). Bacterial CYPs act over many complex substances, which can be useful for the remineralization of the particulate organic matter in the deep ocean. Many *Actinobacteriota* genera, such as *Streptomyces* and *Mycobacterium* are known to harbor many highly diverse CYP enzymes (Senate et al., 2019; Mnguni et al., 2020). An in-depth analysis of encoded *Acidimicrobiales* CYPs revealed that the three deep water-associated genera harbored, on average, a larger number of CYPs in their genomes (5.6 CYPs per 1,000 proteins, SD ± 1.1) than the epipelagic genera MedAcidi-G1 and MedAcidi-G2B (3.0 CYPs per 1,000 proteins, SD ± 1.1; Figure 3B). In addition, the annotation of these proteins in CYP families also revealed that genera S20-B6, UBA3125 and UBA9410 encoded for more diverse protein families, on which 11 CYP families were not detected in the genera of the photic zone (Figure 3B). Although families CYP107, CYP124, CYP125, CYP130, and CYP142, were detected in all genomes of the family, deep-associated genera showed an enrichment in paralogous genes, which may enhance the biodegradation of certain similar compounds (Figure 3B). For instance, CYP102, CYP124, CYP125, and CYP142 families are involved in the catabolism of cholesterol and other sterol derivatives (McLean et al., 2009; Driscoll et al., 2010). In addition, CYP124 and CYP153 are involved in the oxidation of aliphatic hydrocarbons (Johnston et al., 2009). In some cases, one CYP family can harbor different functions. Due to the abundance of the CYP proteins within the *Acidimicrobiales* genomes, we evaluated the presence of these genes in the deep ocean. To that end, we performed a metagenomic search against 28 samples from the Malaspina expedition (Acinas et al., 2021). Normalization of the number of CYPs per 1,000 genes revealed that *Actinobacteriota* was the major contributor of CYPs, followed by *Chloroflexota*, *Alphaproteobacteria*, and *Gammaproteobacteria* in several stations in the Indian, Pacific, and Atlantic oceans and different plankton size fractions, particle-associated (PA) and free-living (FL) (Figure 3C). In addition, we used a vertical profile of samples from the Western Mediterranean Sea to analyze the distribution along the water column. The phylogenetic distribution was analyzed by considering the number of CYPs per 1,000 genes and the abundance normalized by the percentage of 16S rRNA gene reads of each group. Figure 3D shows that *Actinobacteriota* was the group with the highest number of CYPs in all layers of the water column. While reads beyond the deep chlorophyll maximum were assigned to the order *Acidimicrobiales*, in the shallower layers (up to 30 meters depth) they belonged to the other order of pelagic marine bacteria *Actinobacteriota,* “*Ca.* Actinomarinales” (Haro-Moreno et al., 2018). Therefore, these results suggest that marine Acidimicrobiales play an important role in the degradation of recalcitrant compounds throughout the water column.

### Diversity in the regulation of gene expression

Bacteria can sense and respond to external environmental stimuli rapidly, which gives them a great adaptive capacity necessary to survive in environmental changes. This capacity is mainly due to the action of complex regulatory networks carried out by sigma factors, transcriptional regulators, or two-component systems. Among these transcriptional regulators, there is one that is exclusively found in *Actinobacteriota* which is the WhiB-like family of proteins, a unique group of iron–sulfur ([4Fe-4S]) cluster-bound proteins. In model bacteria within *Actinobacteriota* such as *Streptomyces* or *Mycobacteria*, several paralogs have been located in the genome involved in the regulation of virulence, cell division or antibiotic resistance, among others (Bush, 2018). We obtained all the genetic diversity of this transcriptional regulator from the dereplicated genomes by constructing a phylogenetic tree using the sequences of *Mycobacterium tuberculosis* H37Rv and *Streptomyces venezuelae* ATCC10712 as references (Bush, 2018).

The tree showed six major clades, three of them corresponding with reference sequences such as whiB1, whiB2, whiB3, and whiB7 (Supplementary Figure S1A). The whiB4 appears as an independent branch with no homologs in the marine genomes of the MedAcidi-G1 family. The remaining three branches have no homologs in the reference sequences used, and it is noteworthy that one of them consists only of deep-sea-associated genera without any sequences of the genomes associated with the epipelagic zone. This could suggest an important role in deep ocean adaptation (Supplementary Figure S1A). Sequence alignment showed that all sequences possessed the typical four invariant cysteine residues coordinating a [4Fe-4S] cluster, ranging in size from 75 to 153 residues (Supplementary Figure S1B). However, within the exclusive deep clade we found seven sequences with a larger size (*ca.* 246 residues) due to the addition of a tail of about 100 amino acids at the *N*-terminal end (Supplementary Figure S1B).

The presence of a single sequence separate from the marine sequences and located between the WhiB1 and WhiB2 references is noteworthy (Supplementary Figure S1A). This sequence belongs to a MAG (Actinobacteria bacterium isolate NP111, accession number PCBW01000000) that cluster within the genus UBA3125. Contig annotation (PCBW01000032) revealed several viral-related genes with homology to sequences present in the pVOGs (Prokaryotic Virus Orthologous Groups) database (Grazziotin et al., 2017). The viral origin of the sequence was confirmed using VIBRANT software (Kieft et al., 2019) as medium quality draft. Thus, the sequence could belong to a phage of genus UBA3125. The size of the transcription factor WhiB in this sequence is smaller than that of its bacterial counterpart (75 residues) but retains the basic motifs, therefore it can be considered an auxiliary metabolic gene. Host assignment is one of the pending tasks of metagenomics hence this sequence can be used to obtain more viral sequences of this family.

The other type of regulators are sigma factors, which can be divided into two families, σ70 and σ54 (Sun et al., 2017). These regulators are present in all bacteria, most of the factors belong to the σ70 family which is involved in the transcription of housekeeping genes during exponential growth essential for cell survival (Paget and Helmann, 2003). However, the role of σ54 appears to be more limited to nitrogen metabolism (Jones, 2009). While all genera had the σ54, we found differences in abundance in the four subgroups that make up the σ70. The deep-associated genera had a higher amount of σ70 group 1; in *Streptomyces* this group of regulators is involved in the transcription of specific pathway regulatory genes that are essential for the biosynthesis of secondary metabolites (Sun et al., 2017). Among the different two-component systems, we found only one unique to the deep water-associated genera, EnvZ-OmpR. We also found differences in SOS response factors, an inducible DNA damage repair pathway controlled by two key regulators that is not limited to DNA repair. This system is also involved in pathogenesis, biofilm formation, energy metabolism, as well as toxin-antitoxin systems (Podlesek and Žgur Bertok, 2020). While in the surface water-associated genera, we found three types of regulators, RecA (DNA recombination), LexA (DNA recombination) and Ssb (DNA replication, recombination and repair), genomes of the deep water-associated genera had twice as many, with the addition of RecN (DNA repair), ImuB (SOS response) and DnaE2 (SOS response) (Xie et al., 2018).

## Discussion

Due to the technical difficulties of accessing the deep ocean, the study of microbial communities in this environment has been delayed compared to those in the photic zone. In recent years, the widespread use of cultivation-independent techniques in natural microbial communities has advanced our knowledge of these largely unknown, but essential, components of the biosphere, which has been referred to as “microbial dark matter” (Rinke et al., 2013). Our results revealed two different trajectories within the same family of the order *Acidimicrobiales* in their adaptation to different ecological niches within the oceanic water column such as the photic zone and the deep zone of the water column. The different physicochemical and nutritional conditions of both regions would have produced a physical isolation leading to an allopatric speciation (López-Pérez et al., 2019).

This ecological separation seems to have had effects on the genomic architecture. While microbes from the epipelagic zone showed typical traits of streamlined genomes, similar to those found in SAR11 or *Prochlorococcus* (Dufresne et al., 2005; Haro-Moreno et al., 2020), representatives of the aphotic zone had a higher GC content as well as longer intergenic spacers. The variation of GC content with depth has already been suggested as an evolutionary adaptation to the different bioavailability of nutrients between the epipelagic zone and the deep ocean (Mende et al., 2017; Haro-Moreno et al., 2018). Along the same lines, other variables that show how nutrient availability can vary genomic parameters are the N-ARSC and C-ARSC values. The higher N-ARSC and lower C-ARSC values detected in the genomes of the deep water-associated genera are consistent with the higher nitrogen concentration and lower carbon concentration, due to the absence of photosynthesis, in deep waters compared to the photic zone (Getz et al., 2018; Haro-Moreno et al., 2018). Except for genome size, not statistically significant between both groups, similar results (lower GC content and N-ARSC and shorter intergenic regions) were found in the genomic analysis between epipelagic *Marinimicrobia* with their mesopelagic counterparts (Martinez-Gutierrez and Aylward, 2019). Therefore, the convergence of these changes in phylogenetically distant groups could indicate that environmentally imposed nutritional constraints are an important driver of the two observed genomic trends, as has been previously suggested (Mende et al., 2017; Getz et al., 2018; Haro-Moreno et al., 2018; Martinez-Gutierrez and Aylward, 2019).

The different ecological niches and lifestyles (surface and depth) were also reflected in different metabolic capacities. While the presence of rhodopsins in genera adapted to the epipelagic region may supplement the energy metabolism of these microbes, genera from the deeper regions seem to have developed other metabolic strategies with a greater potential for carbohydrate degradation, recalcitrant compounds, as well as the ability to accumulate glycogen as a source of carbon and energy. In addition, genera associated with deeper zones showed an enrichment in the number and diversity of genes encoding elements involved in transcriptional regulation. More complex regulation has been linked to bacteria that show higher metabolic versatility and live in more fluctuating environments (Bervoets and Charlier, 2019). Similarly, differences in regulation between oligotrophic and copiotrophic lifestyles have also been observed in marine environments, where copiotrophic bacteria require more regulation (Cottrell and Kirchman, 2016).

The analysis also revealed an enrichment in CYPs within the entire order *Acidimicrobiales* compared to other well-known degraders of organic matter such as the SAR202 clade within the phylum *Chloroflexota* (Landry et al., 2017). This suggests that these microbes play an important role in the biological oxidation of recalcitrant organic compounds. These results open new avenues for future research in which the contribution of this group to the biochemistry of the dark ocean and its involvement in the global carbon cycle and climate change should be analyzed in detail. Finally, the presence of two distinct ecotypes within the same family makes this group an ideal model for studying microbial adaptations to deep water environments.

## Data availability statement

The original contributions presented in the study are included in the article/Supplementary material, further inquiries can be directed to the corresponding author.

## Author contributions

ML-P and JH-M conceived the study. JH-M, JR-G, and ML-P analysed the data and contributed to write the manuscript. All authors contributed to the article and approved the submitted version.

## Funding

This work was supported by grant “FLEX3GEN” PID2020-118052GB-I00 (cofounded with FEDER funds) from the Spanish Ministerio de Economía, Industria y Competitividad to ML-P.

## Conflict of interest

The authors declare that the research was conducted in the absence of any commercial or financial relationships that could be construed as a potential conflict of interest.

## Publisher’s note

All claims expressed in this article are solely those of the authors and do not necessarily represent those of their affiliated organizations, or those of the publisher, the editors and the reviewers. Any product that may be evaluated in this article, or claim that may be made by its manufacturer, is not guaranteed or endorsed by the publisher.
