# Supplementary figures and images for: Evolutionary pathways for deep-sea adaptation in marine planktonic Actinobacteriota
Source: Front Microbiol. 2023 May 10;14:1159270. doi: 10.3389/fmicb.2023.1159270 (PMC10205998; doi:10.3389/fmicb.2023.1159270)

A

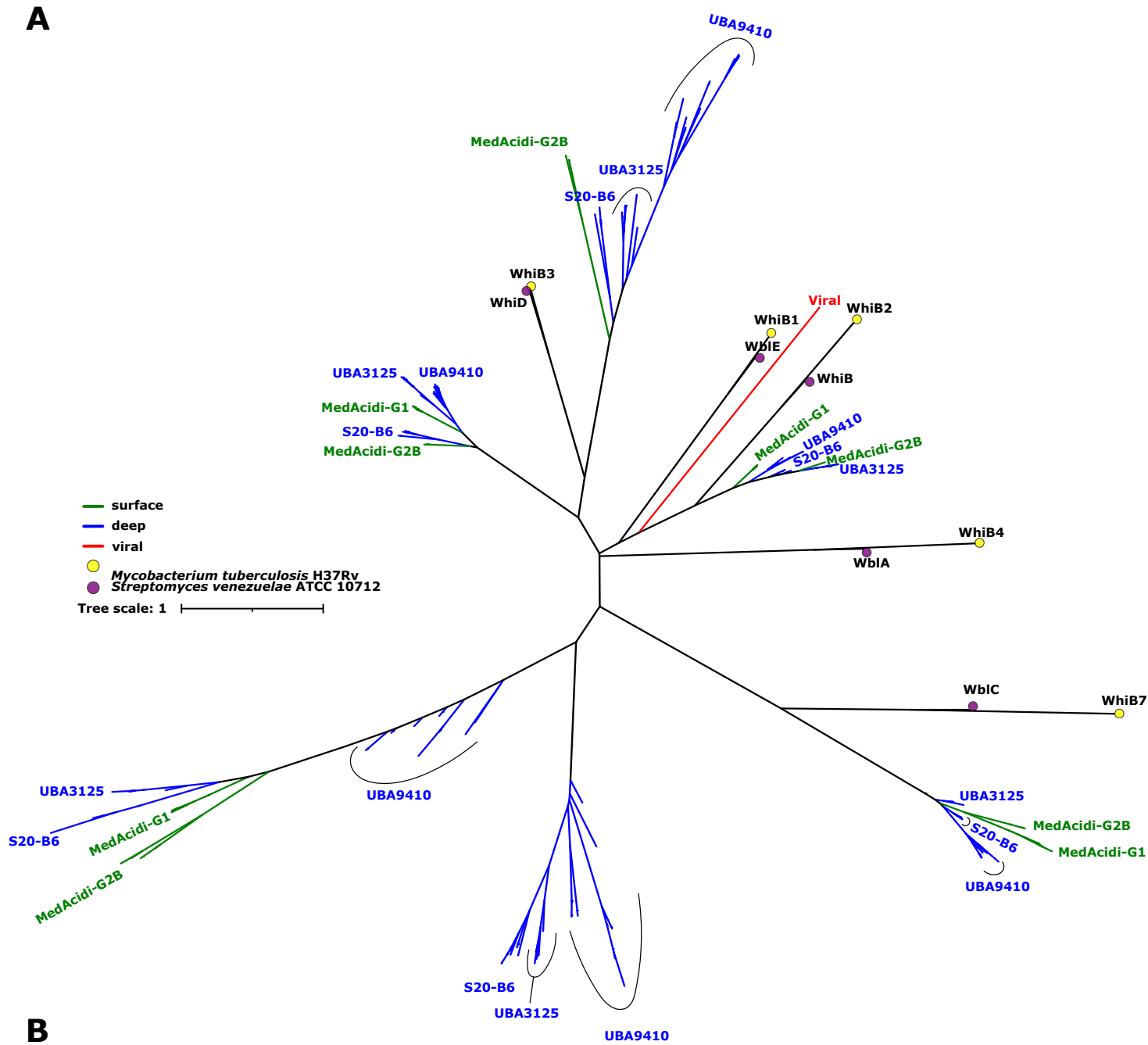

B

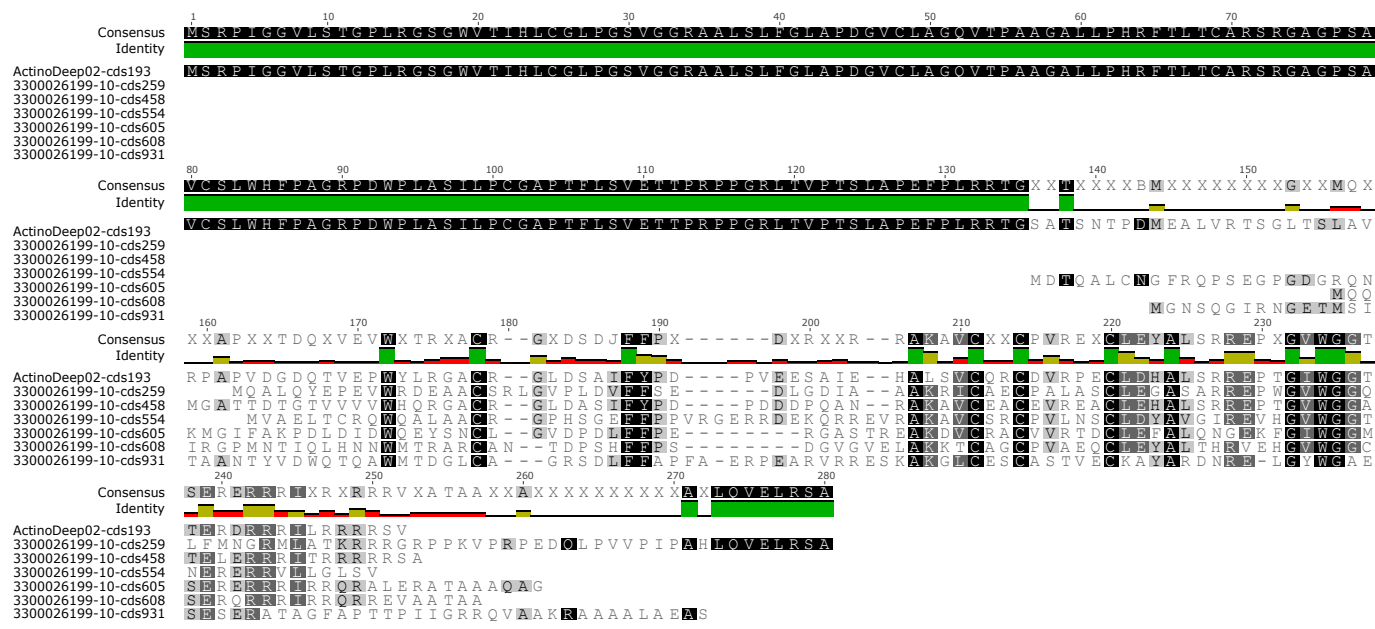

Supplement: Supplementary file 3 [file Data_Sheet_1.PDF]
